# Fire Detection and Notification Method in Ship Areas Using Deep Learning and Computer Vision Approaches

**DOI:** 10.3390/s23167078

**Published:** 2023-08-10

**Authors:** Kuldoshbay Avazov, Muhammad Kafeel Jamil, Bahodir Muminov, Akmalbek Bobomirzaevich Abdusalomov, Young-Im Cho

**Affiliations:** 1 Department of Computer Engineering, Gachon University, Seongnam-si 461-701, Republic of Korea; kuldoshbay@gachon.ac.kr (K.A.);; 2 Department of Artificial Intelligence, Tashkent State University of Economics, Tashkent 100066, Uzbekistan

**Keywords:** fire, flame detection, YOLOv7, deep learning, E-ELAN, ships

## Abstract

Fire incidents occurring onboard ships cause significant consequences that result in substantial effects. Fires on ships can have extensive and severe wide-ranging impacts on matters such as the safety of the crew, cargo, the environment, finances, reputation, etc. Therefore, timely detection of fires is essential for quick responses and powerful mitigation. The study in this research paper presents a fire detection technique based on YOLOv7 (You Only Look Once version 7), incorporating improved deep learning algorithms. The YOLOv7 architecture, with an improved E-ELAN (extended efficient layer aggregation network) as its backbone, serves as the basis of our fire detection system. Its enhanced feature fusion technique makes it superior to all its predecessors. To train the model, we collected 4622 images of various ship scenarios and performed data augmentation techniques such as rotation, horizontal and vertical flips, and scaling. Our model, through rigorous evaluation, showcases enhanced capabilities of fire recognition to improve maritime safety. The proposed strategy successfully achieves an accuracy of 93% in detecting fires to minimize catastrophic incidents. Objects having visual similarities to fire may lead to false prediction and detection by the model, but this can be controlled by expanding the dataset. However, our model can be utilized as a real-time fire detector in challenging environments and for small-object detection. Advancements in deep learning models hold the potential to enhance safety measures, and our proposed model in this paper exhibits this potential. Experimental results proved that the proposed method can be used successfully for the protection of ships and in monitoring fires in ship port areas. Finally, we compared the performance of our method with those of recently reported fire-detection approaches employing widely used performance matrices to test the fire classification results achieved.

## 1. Introduction

Around 71% of the Earth’s surface is covered by oceans, and the enormous water area produces natural canals [1]. Ships are the oldest means of transportation and have helped mankind in various matters of life by using water. Since the 15th century, the fast expansion of shipping has made it possible for humans to move between continents with the main purpose of transporting goods and travelers, and the massive exchange of personnel and things has drastically affected the social and natural landscape. Ships have evolved with the passage of time. Advancements in technology have brought a whole new potential to the shipping experience and made it more reliable for humans to work through sea routes.

While shipping is the most effective use of transportation, it also brings major hazardous threats to the lives on ships. Among all the dangers that come with the shipping experience, the threat of fire is one common occurrence. Passengers and crew onboard are more exposed to danger if the cargo includes highly inflammable material such as oil, gas, coal, or wood. If any of these items catch fire, the results would be catastrophic. Additionally, a fire accident on a ship is highly likely to be fatal to human life because it is difficult to receive fire suppression support from the outside due to the nature of the closed and isolated space of the sea, and it is necessary to extinguish the fire with a limited amount of personnel and equipment [2].

The key factor which results in escalating ship fires is a lack of awareness and prior safety measures. Having no fire prevention and detection measures leads to some unpleasant incidents. Hence, installing such an efficient and reliable system to detect a fire at its early stage is crucial.

To prevent the expansion of a fire, two methods are commonly used:

(1) Traditional fire alarm system;
(2) Fire detection system based on computer vision.

A fire alarm system comprises physical sensors such as flame detectors, thermal detectors, smoke detectors, etc. The main drawback of this type of system is that it involves human intervention to check and approve the evaluation of a fire. In addition, it uses a variety of tools to detect the fumes, smoke, and intensity of a fire. These sensors can detect a fire when it evolves, and fumes and smoke lead to flames, but it is risky to let a fire expand to such an extent that it can lead to serious damage. A 10 min delay in putting out a fire in the engine room may cost USD 200,000, while a 20 min delay may cost up to USD 2,000,000 [3].

The alternative to fire alarm systems is AI-based fire detection. Lately, the use of deep learning algorithms has found its way into detecting fires through images. Recent research has proved the effectiveness of computer vision- and deep learning-based methods for fire detection [4,5]. Deep learning target detection can automatically extract image details and features, effectively overcoming the redundancy and interference caused by the manual extraction of image features for fire detection [6]. Fire detection is a more challenging task and needs proper, up-to-date technology to alert the crew. To overcome this issue, one of the most popular OS projects in computer vision is used, named YOLO (You Only Look Once). YOLO is an efficient real-time object detection algorithm that divides an image into a grid system, and each grid detects objects within itself. It can be used for real-time inference and requires very few computational resources [7]. YOLOv6 has improved object detection accuracy, particularly for recognizing small objects. YOLOv6 is an innovative system for real-time object detection based on deep learning [4]. However, due to more power consumption and being computationally expensive in nature, it comes with some limitations, which were addressed in YOLOv7. YOLOv7’s basic premise is to enhance detection accuracy and performance while simultaneously minimizing the number of parameters and amount of processing required. However, when it comes to the detecting layer, YOLOv7 employs not one, but two heads: the lead head and the auxiliary head. Because of their interaction, these two layers give a more detailed portrayal of the data’s correlation and distribution [4]. Fire detection is the most challenging process because of ambiguous nature of it; the following are some of the most important advantages of the proposed strategy:

i. We will publish a dataset for fire detection that will be used to detect fires in both daytime and nighttime scenarios. Fire and flame predictions will be précised, and overfitting will be minimized as a deep CNN (convolutional neural network) learns from a vast database of fire detection images.
ii. We provide a YOLOv7-based active method of fire detection to strengthen the protection and to eschew long operations.
iii. While rotating fire datasets by 25 degrees, a mechanism was devised to mechanically reorder flagged containers.
iv. During the training phase of YOLOv7, class predictions were generated utilizing independent logistic classifications and a binary cross-entropy loss. This is far faster than other detecting networks [4].
v. In order to decrease the number of false positives in the fire recognition method, we utilized photos that resembled fire and excluded low-resolution photographs. Additionally, even in tiny fire zones, the suggested approach considerably improves accuracy and lowers the percentage of false detections.

YOLOv7 incorporates several new features, including a modified backbone network, improved feature fusion techniques, and more efficient training and inference processes. These modifications result in improved accuracy and faster inference times as compared to YOLOv4. In particular, the YOLOv7 model has shown superior performance in detecting small objects, making it well-suited for the detection of fires on ships, which are often localized and relatively small.

## 2. Related Work

Most of the object-detecting and object-recognizing algorithms depend on a particular type of deep neural network (DNN) and CNN. Learnable neural networks comprise various layers to perform object detection. Each layer is responsible for performing different tasks such as analyzing the areas, extracting features of the data obtained, identifying data, and detecting any anomalous behavior. Traditional fire detection methods were lacking in speed and accuracy and suffered from performance degradation. Deep learning fire detection techniques have emerged in the past decade, among which YOLO algorithms have aided in solving the major object detection problems. Development of YOLO’s framework is highly based on improvements in the upcoming models. From the original YOLOv1 to the latest YOLOv8 algorithms, the model’s performance with key innovations and differences has evolved to accomplish detection tasks.

Although YOLOv6 and YOLOv7 have their unique features and limitations, they share some common traits. For instance, they use deep convolutional neural networks as the backbone architecture, adopt a one-stage object detection paradigm, and employ modern optimization techniques such as batch normalization and adaptive moment estimation. However, they also differ in some respects; for example, YOLOv6 and YOLOv7 use anchor-based prediction and anchor-free prediction, respectively. YOLOv7, on the other hand, is a more lightweight version of YOLOv6 that addresses the computational and memory limitations of YOLOv6 [8]. YOLOv7 has shown promising results in fire detection, but it needs to be trained more accurately for rare situations like a fire in the engines. Its high accuracy, speed, and ability to detect small objects make it well-suited for the task at hand. The following are the related state-of-the-art works in the field of fire detection and object detection.

### 2.1. Traditional Fire Detection Techniques

A typical fire detection system onboard a ship involves sensors (fire/smoke/heat) and an alarm panel [9]. Fire detectors are designed to provide a visible and audible alarm on the vessel to indicate the location of a fire. The detectors throughout the ship are wired to a fire control panel that provides visual and auditory alerts and possibly alarms in other parts of the vessel as well. The authors in [10] proposed a ship fire monitoring and alarm system using CAN bus technology. Some types of detectors may sense a rapid rise in temperature in a brief period and then alert the ship, while others may detect fires on a visual basis such as smoke or fire on a camera system to set off said alarm. Traditional fire detection systems involve the need for physical sensors that require human intervention to confirm the occurrence of a fire. Several other tools are incorporated with sensing devices to detect fire, flames, and smoke. However, these detectors are inefficient, as they cannot distinguish between smoke and fire, thus resulting in false alarm generation.

### 2.2. Different Fire Detection Methods Using Deep Learning Algorithms

With the growth of AI, numerous research attempts have been made to detect the presence of fire/smoke in images using machine learning and deep learning models. In a range of computer-based vision applications, such as visual recognition and image classification, the introduction of CNNs has resulted in significant performance gains [11]. The convolutional neural network (also known as CNN or ConvNet) is one of the most popular deep neural networks in deep learning, especially when it comes to computer vision applications. It uses a special technique called convolution. In [12], detection of fire and smoke through images and videos using deep learning algorithms is performed, including a CNN-based architecture to train a model with many images for a dataset. Dilated convolutional layers have been built to avoid depth of learning, which means to learn larger data by ignoring the minute details. Classifying smoke and fire to reduce false fire alarms is accomplished successfully in this research work. Differentiation between smoke and fire in images and videos is accomplished by using a dilated CNN to learn the robustness of features from the scene. The experimental results indicate that the proposed method performs slightly better than well-known neural network architectures on their custom dataset. However, the main limitation is that errors occur when pixel values come closer to those of background and edges or are not detected by the CNN. Because it is a custom-built dataset, it is computationally expensive. In [13] a deep learning-based fire detection system called Detection and Temporal Accumulations (DTA) is used that imitates the human detection process to improve the accuracy of fire detection while reducing false detections and misinterpretations. The proposed method successfully interprets the temporal SRoF behaviors and improves the fire detection accuracy. The faster R-CNN model is used, which can detect multiple objects in a frame. It can detect fire, flames, and smoke in a frame. Long short-term memory (LSTM) to accumulate the temporal behaviors and to decide whether there is a fire or not in a short-term period is also used. In [14], the authors designed a lightweight convolutional neural network for early fire and smoke detection which successfully achieved competitive accuracy. It uses two satellite imagery datasets and three smoke-related scene classes, namely, “Smoke”, “Clear”, and “Other aerosol”. This model needs more improvements, as some of the smoke patches were misinterpreted as “Clear” or “Other aerosol”, which is troubling for the early prediction of fires [14].

### 2.3. Fire Detection Using YOLO (You Only Look Once) Algorithms

In [15], the YOLOV3 algorithm is used for a small-scale flame detection method. This method was proposed to achieve the detection of different scales of flames using an improved K-means clustering algorithm. The authors of [16] introduce a fire-YOLO algorithm. It adds depth-separable convolution to YOLOv4 and helps to reduce the computational costs of the model and improve the perceptual field of the feature layer by using a cavity convolution method. The authors of [17] proposed a fire detection technique for urban areas using ELASTIC-YOLOv3 as an improvement on YOLOv2 to amplify the performance without introducing more parameters. Traditional fire algorithms, especially the ones for nocturnal fire detection, suffered from issues like high light intensity, lack of color information, changes in shapes and sizes of flames, etc., for which more advanced and improved real-time fire detection and recognition systems came with modified YOLO algorithms (v4, v5), as proposed in [18,19].

## 3. Proposed Work

### 3.1. Fire Dataset Description

**Figure 1:**
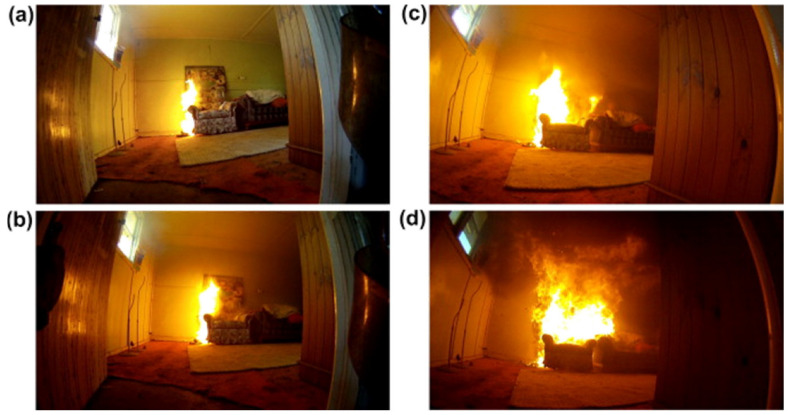
Ship room example images of fire dataset (**a**–**d**).

**Figure 2:**
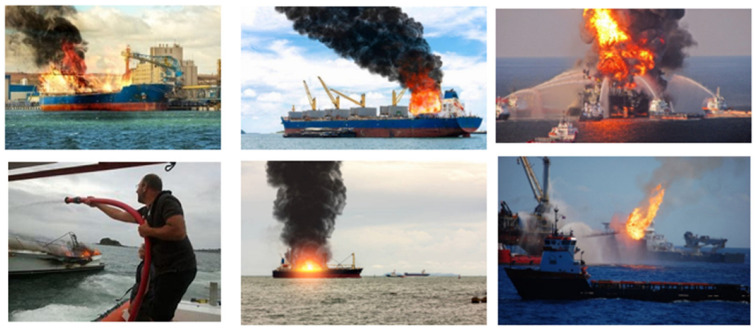
Ships in fire dataset.

To train the model, we collected a diverse range of images of fires from various internet sources, including some videos to increase the size of dataset. For this purpose, images obtained from distinct angles, focal lengths, and brightening conditions were utilized in our dataset to elevate the accuracy of the system. Even after exploring different resources, the images were not enough for the dataset, so additionally, images from publicly accessible dataset platforms such as Roboflow and Kaggle were included to broaden the dataset, as shown in Table 1. The illustration features fire items, flames, and burning displays. The dataset’s diversity strengthens the model’s ability to generalize unseen or unexpected data and adapt to changing conditions. Containing both diurnal and nocturnal images, our dataset reached a total of 4186 images of fires (Figure 1 and Figure 2).

Another 436 images of non-fire scenarios were added to expand the diversity of the dataset for more accurate results. Altogether, the dataset contains 4622 images from both the fire and non-fire categories (Table 2). They are divided as follows: 70% for training, 10% for testing, and 20% for validation. For the test dataset, we tried to accumulate as many realistic images as possible because the fire detection unit must ultimately work in these situations only. Because our training dataset is already diverse enough, we used these realistic images for testing purposes only [20].

Due to several circumstances, including a known common component, there is a high likelihood of overfitting, including a lack of data to adequately capture all potential input conditions. Applying data augmentation techniques to expand the training dataset is an efficient way to combat overfitting. Augmentation includes applying different geometric transformations and distortions to the images such as scaling, rotation, random crops, vertical flipping, horizontal flipping, and contrast enhancement to increase the variety of images for model training and improve the determined accuracy. The size and resolution of the images directly impact the efficiency of the trained model. In addition, one of the biggest problems in object detection is different weather conditions or low model performance in some situations (sun reflection, lack of light, etc.) [21]. Therefore, it is significant to apply data augmentation techniques to expand the dataset for model training [22,23,24,25]. By applying data augmentation techniques to the images, we perform rotation and scaling on each image, which doubled the number of obtained images. The aim is to train the model to detect fires, which requires the use of a large number of fire images to enhance the performance. Augmenting the data makes it possible for the object detection system to detect and recognize objects from different perspectives to achieve the maximum performance and accuracy of the model.

Setting up the original dataset during data collection is followed by data annotations. It is an important step resulting in efficient performance of the trained model. If the bounding boxes around the classes of interest were too loosely defined, this could force the models to generalize on a false assumption. Conversely, very stringent bounding boxes could result in missing a section of the relevant class, again leading to the risk of false generalization during training [3]. To surmount these two risks, we used data annotations based on closed proximity.

### 3.2. Methodology

YOLO models not only perform object detection and recognition but are also used for instance segmentation, semantic segmentation, and pose tracking by dividing images into a grid and creating bounding boxes around predicted probabilities of classes in each cell of the grid. The evolution of YOLO models is typically based on the accuracy and speed of the algorithm. The YOLOv7 method has much better performance and has achieved great success in the 5 FPS-to-160 FPS range, exceeding the speed and accuracy of currently known target detectors [26].

YOLOv1, the original and earliest YOLO model, was introduced to detect real-time objects but was lacking in speed and detection of small objects. YOLOv2 helped in achieving faster inference times with more advanced extraction of features, but anchor boxes were of the same size for every object. YOLOv3 brought advancement by allowing scaled anchor boxes for the respective sizes of objects, along with improved speed and accuracy, but suffered in detecting small objects and had higher memory requirements. With advanced data augmentation techniques, YOLOv4 brought improvements to maintain real-time performance. While it is common to compromise on accuracy to improve the speed of a model, YOLOv5 came with the aim of maintaining accuracy with an increase in speed by using advanced techniques of training such as focal loss, label smoothing, and multi-scale training. YOLOv6 successfully achieved stronger performance against the MS COCO dataset. It is more efficient than all previous versions of YOLO in terms of accuracy. It has introduced a new technique to generate boxes called “Dense anchor boxes”.

**Figure 3:**
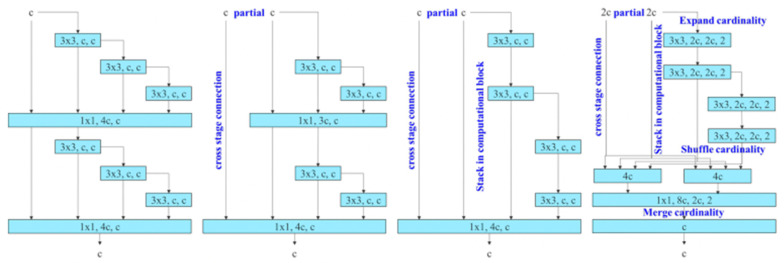
E-ELAN final aggregation layer and an extended version of the ELAN computational block.

YOLOv7, on the other hand, outperforms its predecessors with an efficient backbone network (E-ELAN) and an improved feature fusion technique (Figure 3). For the detection and recognition of small objects, YOLOv7 is preferrable to YOLOv6 in compound environments. YOLOv7 proposed a couple of architecture changes and a series of bag-of-freebies methods, which increased the accuracy without affecting the inference speed, only the training time [27]. It combines an attention mechanism and a re-parameterization convolutional structure [28]. YOLOv7 is also inspired by re-parameterized convolutions (RepConv), just like YOLOv6. It amalgamates different convolutional modules into a single inference degree. This technique is split into two categories: the model-level ensemble, which trains multiple models of the same nature with different training data, and the module-level ensemble technique, which has gained more popularity due to its performance as a weighted average on the weights of models at various iterations. However, some of the re-parameterization techniques are architecture-specific, meaning they only work with specific architectures. YOLOv7 is a solution to overcome the previous method’s drawbacks. It utilizes gradient flow propagation paths for determining the segments (modules) within the overall model that requires re-parameterization [29].

YOLOv7 uses an E-ELAN architecture as the backbone of the algorithm. E-ELAN uses expand, shuffle, and merge cardinality methods to achieve the ability to continuously enhance the learning ability of the network without destroying the original gradient path [8].

**Figure 4:**
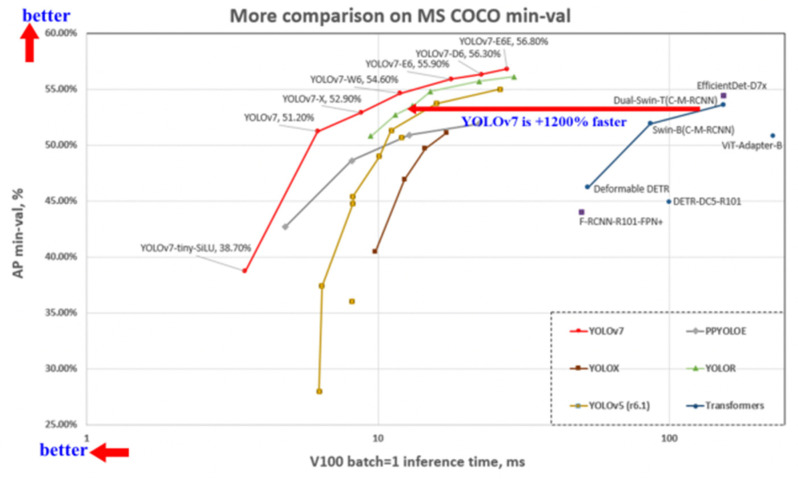
YOLOv7 evaluates in the upper left—faster and more accurate than its peer networks [30].

Different models of YOLOv7 include YOLOv7, YOLOv7-W6, YOLOv7-tiny, YOLOv7-X, YOLOv7-E6, and YOLOv7-D6. YOLOv7 is a basic model for ordinary GPU computing. YOLOv7-W6 is optimized for cloud GPU computing. YOLOv7-tiny is used for edge GPUs, while YOLOv7(X, E6, D6) are obtained from a compound scaling method. A major advantage of YOLOv7 over its antecedents is its speed and accuracy, which empower it to perform object detection more precisely and accurately, as shown in Figure 4. Unlike other state-of-the-art algorithms, YOLOv7 processes images at a speed of 155 frames/second, which is much faster than the earlier versions. It achieves 37.2% as its IoU on the MS COCO dataset. A comparison between the average precision (AP⁵⁰) values of different variants of YOLOv7 using the MS COCO dataset is given in Table 3.

### 3.3. Fire Detection Using YOLOv7

Among various metrics for evaluation such as average precision (AP), F1 scores, recall, and mAP, intersection over union (IoU) was used specially to demonstrate YOLOv7’s object detection capacity. IoU is the measure of the amount of overlap between the detected object and the ground truth object [32]. The general equation for IoU is given as:

(1) $$\mathrm{IoU}=\frac{\mathrm{Area}\mathrm{of}\mathrm{intersection}}{\mathrm{Area}\mathrm{of}\mathrm{Union}}$$

**Figure 5:**
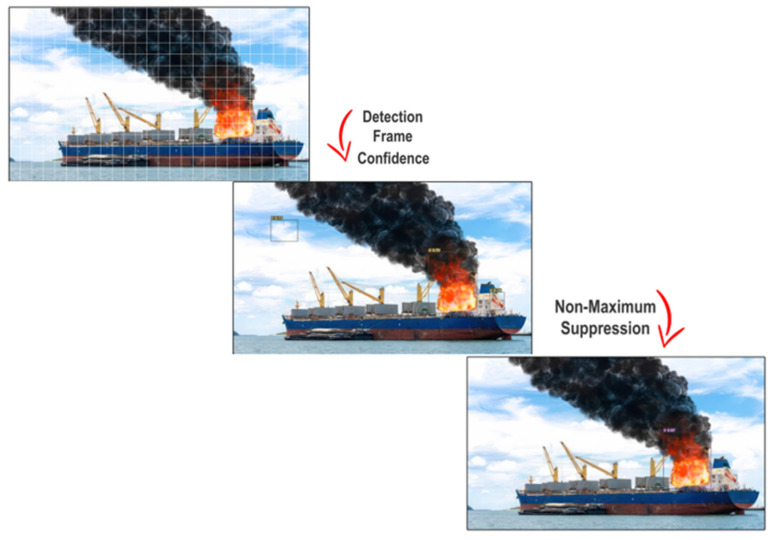
Detection by YOLOv7 using NMS.

It is standard to use IoU to gain insights about a model’s overall performance in terms of localization. The YOLOv7 model performs object detection in a single stage. First, it separates the input image into N grids, all of same size. Every region of the image is analyzed to detect the classified object. In each grid, objects are predicted with bounding boxes along with their label and probability score to read the potential object’s presence. Predicted objects in each grid are overlapped from the increasing predictions of the grid; thus, redundancy occurs. The YOLO architecture uses a mechanism to predict only objects of interest, called non-maximal suppression. For this, all those bounding boxes predicted with low probability scores are suppressed by comparing the decision with those of the largest probability score. Bounding boxes with largest intersection over union (IoU) with the highest-probability box are removed. This iteration continues until the desired box of highest probability is found, as shown in Figure 5.

Object detection models require knowledge about the depth of the network, width of the network, and resolution of the trained network. YOLO, along with other object detection models, uses single-dimensional scale methods with high human adaptation, which could not scale up a desired dimension without changing the input and output channel of a transition layer. However, YOLOv7, unlike its peers, scales the depth and width of the network simultaneously while connecting layers together. This mechanism of YOLOv7 preserves the optimization of the model while scaling for various sizes.

## 4. Experimental Results and Discussion

### 4.1. Model Evaluation

We implemented and tested our model using Visual Studio 2022 C++ on our laptop with a CPU speed of 3.20 Hz, 32 GB RAM, and 3 GPUs. To test our ship fire detection model, we implemented it in different environments. The dataset was collected using various resources on internet and annotated in YOLO form. Both the YOLOv6 and YOLOv7 models were trained by setting suitable values of different parameters. Pytorch, a deep learning framework, was used to train the model with Google Colab Pro, an Nvidia A100 GPU, and 48 GB of memory, and various tests were conducted to validate the performance and effectiveness of the trained models. There are some metrics, such as accuracy, recall, F1 score, AP, and mAP, that are crucial to determine the validity of models. These are evaluation parameters in object detection processes. Accuracy is the closeness of a quantity’s measurement value to its actual value. The ratio of true positives (TP) to all predicted positives is precision. Recall is the ratio of true positives (TP) to all ground truths. F1 score is calculated as the harmonic mean of the precision and recall values [33], which indicates better target detection accuracy [34]. The F1 score ranges between 0 and 1, with a higher value indicating better model performance, as detailed in these papers [35,36,37,38,39,40].

(2) $$\mathrm{Accuracy}=\frac{\mathrm{TP}+\mathrm{TN}}{\mathrm{TP}+\mathrm{TN}+\mathrm{FP}+\mathrm{FN}}\;$$

(3) $$\mathrm{Precision}=\frac{\mathrm{TP}}{\mathrm{TP}+\mathrm{FP}}$$

(4) $$\mathrm{Recall}=\frac{\mathrm{TP}}{\mathrm{TP}+\mathrm{FN}}$$

(5) $$\mathrm{FM}=\frac{2\times \mathrm{precision}\times \mathrm{recall}}{\mathrm{precision}+\mathrm{recall}}$$

TP, TN, FP, and FN are terminologies used to represent the outcomes of a classification model’s predictions compared to the ground truth labels. A true positive (TP) is the number of pixels belonging to a fire detected as positive by the model which the ground truth also labels as positive. A true negative (TN) is the number of pixels belonging to a non-fire detected as negative by the model which the ground truth also labels as negative. A false positive (FP) is the number of pixels classified as a fire detected as positive by the model but which the ground truth labels as negative. A false negative (FN) is the number of pixels classified as a non-fire detected as negative by the model but which the ground truth labels as positive (Table 4).

**Figure 6:**
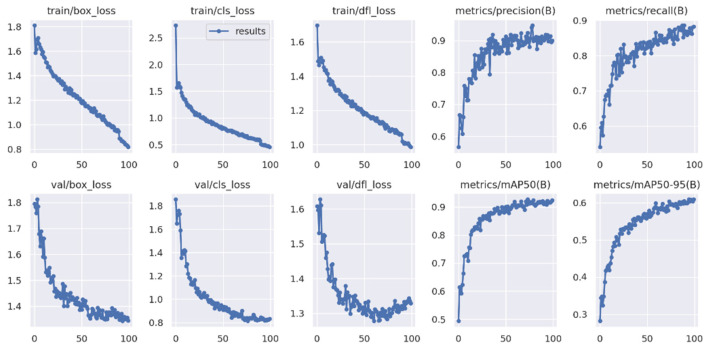
Model evaluation matrices curves.

The mAP is the mean AP used to measure the general detection accuracy of the target detection algorithm. In summary, for the YOLO algorithm, the AP and mAP are the best metrics to measure the detection accuracy of the model [41] as shown in Table 5. Figure 6 shows model evaluation matrices curves. Mean average precision can be described as follows:

(6) $$\mathrm{mAPi}=\overset{1}{\underset{0}{\int }}\mathrm{Pi}\mathrm{Ri}\mathrm{dRi}$$

### 4.2. Analysis by Experiment

The experimental objective was to estimate the performance and concreteness of the system in effectively detecting fires on a ship by consolidating safety and early response abilities. To elevate the system’s robustness and reliability, experiments were conducted in various defined conditions. We used a dataset of distinctive images of fires and flames from different sources, including publicly available datasets. The dataset was then annotated with bounding boxes, and labels were added. A high-performance platform along with an appropriate GPU was used for training. The evaluation of the model depends upon several performance metrics to measure the predicted probabilities correctly. These include precision, recall, F1 score, and accuracy to evaluate the overall detection performance.

**Figure 7:**
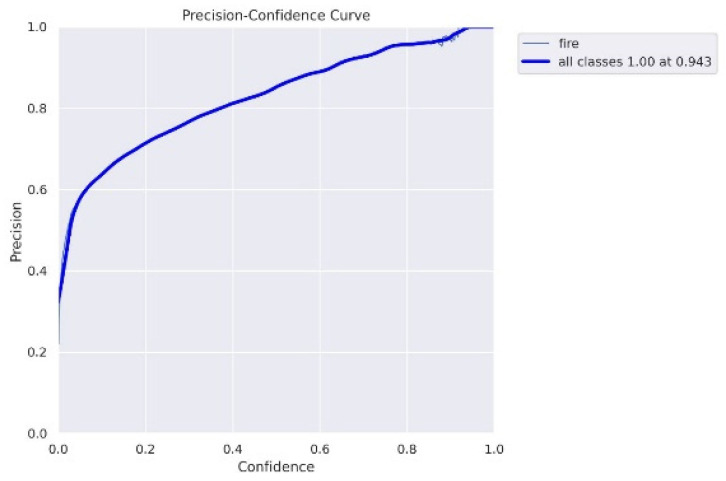
Precision–confidence curve of our model.

Our fire detection system has achieved a precision of 94%. This implies that 94% of fire instances were indeed actual fires, minimizing the rate of false positives lowering the chances of false alerts and alarms. The recall factor of the system was found to be 90%, implying that the system is sensitive enough to recognize and detect fires. The F1 score of the system is estimated to be 86%, which shows a balanced trade-off between the recall and precision, resulting in the overall efficiency of the system in detecting true fires. The proposed fire detection system accomplished an accuracy of 93%, which indicates that fire instances are detected correctly by the system in the test dataset. The experimental results indicate that the fire detection system for ships utilizing the YOLOv7 model performed effectively in detecting fires on ships. The high accuracy, precision, and recall values sum up the system’s robustness and reliability in identifying fire and flames. The obtained F1 score further clarifies the model’s accurate detection of fires. The precision–confidence curve is shown below in Figure 7.

The precision–confidence curve indicates the confidence score at each precision value. The above plot visually represents variations in precision of the detected fires with respect to confidence threshold set for fire detection. When the confidence threshold is set to as low as 0.1, the precision obtained for our model is 0.94, which illustrates that the detection of true positives is relatively high, and the system is efficient enough in detecting actual fires.

### 4.3. Performance of Model in Varying Ambient Lighting

**Figure 8:**
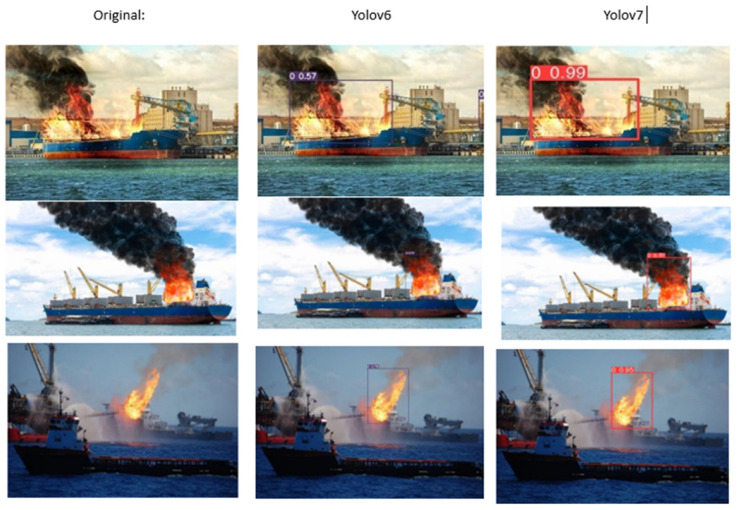
Results of fire detection system for day images.

**Figure 9:**
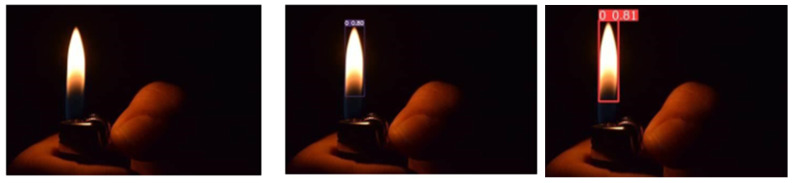
Results of fire detection system for night images.

The system is tested in real-world scenarios with varying light conditions. The model is not only trained for daylight fire detection, but is also trained for situations where the lighting is comparatively darker. Hence, through experiments, the system responds well, performing in both day and night lighting conditions, which makes it more reliable. Figure 8 and Figure 9 illustrate fire detection in different lighting conditions.

### 4.4. Discussion

The success of the novel system is due to the YOLOv7 model’s ability to localize fires and differentiate between fire and non-fire objects. The contribution of deep learning techniques united with the diverse dataset of 4622 images of 640 × 640 size has an immediate impact on the system’s strong implementation. It is worth noting that a system’s efficiency and performance may vary in various conditions such as lighting variations, smoke, variable sensitive environmental factors, and obstruction. Compared to other YOLO models, YOLOv7 has given higher-class results in detecting fires in bright and dark lighting, recognizing small fires and flames, and distinguishing fire and non-fire scenarios. The early detection of fires in ships is now improved with the proposed YOLOv7 system in real time.

Depending on weather, reflection, darkness, and sunlight, actual ship fire images can be dark and blurred. Table 6 compares the recently published fire detection methods with the proposed method.

## 5. Limitations

**Figure 10:**
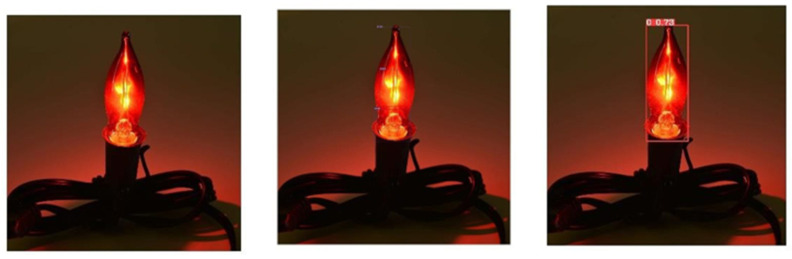
Detection of fire-like bulb as fire.

This research study proposed a fire detection system for ships utilizing the YOLOv7 algorithm which exhibits high performance and accuracy in the detection of ship fires. However, there are some limitations of the proposed strategy which bound its performance. It was observed during experimentation that some of the images containing fire-like objects were recognized as fire. If an image contains bright sunlight, intense yellowish red lights, or fire-like bulbs, then it will be detected as a fire, as shown in Figure 10. Moreover, the model detects red light with high illuminance as fire. These issues could be resolved by expanding the size of the dataset. We aim to retrain our model with a more diverse dataset to overcome these issues [45,46,47].

## 6. Conclusions

In conclusion, this study proposed an improved and faster version of a fire detection system for ships using the YOLOv7 architecture. The thorough experiments and evaluation of the system demonstrate that the proposed system is highly efficient in detecting real-time fires in challenging environments. Our system’s methodology comprises collecting a dataset with vast number of images of various fire scenarios and preprocessing of the collected dataset, which includes data augmentation techniques and model training. The evaluation of the model is compared with existing fire detection systems, and the results indicate that YOLOv7 exhibited high accuracy and ability to detect fires.

The obtained mAP indicates that the achieved performance of our trained model, using the YOLOv7 architecture, is highly effective and can be utilized for detecting real-time fires in maritime environments. Implementation of this model provides timely responses, allowing the mitigation of fires before they escalate. There is a significant contribution of YOLOv7 in leveraging the power of deep learning techniques to timely classify fire instances and prevent fire expansion. YOLOv7 outperforms its peers in small-target detections and escalated our model’s capability of detecting minor fires.

Despite being able to minimize the risks of potential hazards and detecting fires at early stages, there are still areas of improvement. Our proposed system suffers in detecting fire smoke and lacks in detection of fire when there is a comparatively low level of ambient light. Future work on our model could emphasize broadening the dataset and including more images of fire scenarios and other fire-related factors to enhance the adaptivity of the model in various environments. Moreover, smoke detection by the system can be added to expand the implementation areas of the model.

## Figures and Tables

**Table 1:** Allocation of fire images in the dataset.

Dataset	Size	Open-Source Images	Video Frames	Total
Fire Images	640 × 640	1586	2600	4186

**Table 2:** Distribution of fire images in the dataset.

Dataset	Training Images	Testing Images	Validation Images	Total
Fire	2931	418	837	4186
Non-Fire	306	43	87	436

**Table 3:** Different Variants of Yolov7 [31].

Model	Test Size	AP Test	AP50 Test	AP75 Test	Batch 1 fps	Batch 32 Average Time
YOLOv7	640	51.40%	69.70%	55.90%	161 fps	2.8 ms
YOLOv7-X	640	53.10%	71.20%	57.80%	114 fps	4.3 ms
YOLOv7-W6	1280	54.90%	72.60%	60.10%	84 fps	7.6 ms
YOLOv7-E6	1280	56.00%	73.50%	61.20%	56 fps	12.3 ms
YOLOv7-D6	1280	56.60%	74.00%	61.80%	44 fps	15.0 ms
YOLOv7-E6E	1280	56.80%	74.40%	62.10%	36 fps	18.7 ms

**Table 4:** Showing predicted vs. original labels.

Terminology	Ground Truth	Predicted
TP	Positive	Positive
FN	Positive	Negative
FP	Negative	Positive
TP	Negative	Negative

**Table 5:** The result of the model validation matrices.

Acc (%)	*p* (%)	R (%)	FM (%)	mAP50 (%)
0.93	0.94	0.93	0.93	0.81

**Table 6:** Quantitative results of fire detection.

Algorithm	*p* (%)	*R* (%)	*FM* (%)	*Average* (%)
Dilated CNNs [12]	98.9	97.4	98.2	**98.1**
Detectron3 [20]	99.3	99.4	99.5	**98.9**
LCNN [42]	98.7	94.5	97.2	**98.5**
AlexNet [43]	73.3	61.3	75.1	**69.9**
Faster R-CNN [44]	81.7	94.5	87.2	**87.8**
**Proposed Method**	94.3	93.4	94.5	**94.1**

## Data Availability

Not applicable.
